# 3D Unsupervised deep learning method for magnetic resonance imaging-to-computed tomography synthesis in prostate radiotherapy

**DOI:** 10.1016/j.phro.2024.100612

**Published:** 2024-07-19

**Authors:** Blanche Texier, Cédric Hémon, Adélie Queffélec, Jason Dowling, Igor Bessieres, Peter Greer, Oscar Acosta, Adrien Boue-Rafle, Renaud de Crevoisier, Caroline Lafond, Joël Castelli, Anaïs Barateau, Jean-Claude Nunes

**Affiliations:** a Univ. Rennes, CLCC Eugène Marquis, INSERM, LTSI – UMR 1099, F-35000 Rennes, France; b CSIRO Australian e-Health Research Centre, Herston, Queensland, Australia; c Centre Georges-François Leclerc, Dijon, France; d Univ. of Newcastle, School of Mathematical and Physical Sciences, Dept. of Radiation-Oncology Calvary Mater Hospital, Newcastle, Australia

**Keywords:** cGAN, Synthetic CT, Unsupervised learning, Perceptual loss

## Abstract

**Background and purpose:**

Magnetic resonance imaging (MRI)-to-computed tomography (CT) synthesis is essential in MRI-only radiotherapy workflows, particularly through deep learning techniques known for their accuracy. However, current supervised methods are limited to specific center’s learnings and depend on registration precision. The aim of this study was to evaluate the accuracy of unsupervised and supervised approaches in the context of prostate MRI-to-CT generation for radiotherapy dose calculation.

**Methods:**

CT/MRI image pairs from 99 prostate cancer patients across three different centers were used. A comparison between supervised and unsupervised conditional Generative Adversarial Networks (cGAN) was conducted. Unsupervised training incorporates a style transfer method with. Content and Style Representation for Enhanced Perceptual synthesis (CREPs) loss. For dose evaluation, the photon prescription dose was 60 Gy delivered in volumetric modulated arc therapy (VMAT). Imaging endpoint for sCT evaluation was Mean Absolute Error (MAE). Dosimetric endpoints included absolute dose differences and gamma analysis between CT and sCT dose calculations.

**Results:**

The unsupervised paired network exhibited the highest accuracy for the body with a MAE at 33.6 HU, the highest MAE was 45.5 HU obtained with unsupervised unpaired learning. All architectures provided clinically acceptable results for dose calculation with gamma pass rates above 94 % (1 % 1 mm 10 %).

**Conclusions:**

This study shows that multicenter data can produce accurate sCTs via unsupervised learning, eliminating CT-MRI registration. The sCTs not only matched HU values but also enabled precise dose calculations, suggesting their potential for wider use in MRI-only radiotherapy workflows.

## 1 Introduction

While computed tomography (CT) is commonly used for radiation therapy (RT) treatment planning, magnetic resonance imaging (MRI) offers better soft tissue contrast and enables more accurate target volume visualization and delineation [1]. With the increasing use of MR-linac devices [2], MR-only RT workflows have gained increasing attention. In these workflows, MR images from dose planning could be used to estimate the delivered dose (through dose accumulation). However, MRI does not provide electronic density information required for accurate dose calculation [3]. This is why the generation of synthetic CTs (sCTs) from MRI has been proposed [4], [5]. The literature underlines that the most efficient methods use deep learning (DL) [6]. Furthermore, several studies [7], [8] have highlighted a large variability in images across care centers, including variations in MR sequence, resolution, field of view (FOV), and magnetic field strength, which can decrease the robustness of DL models. An alternative strategy suggested to enhance model robustness is the implementation of multicenter training, as discussed in [8], [9], [10], [11], [12].

However, the application of this approach in the pelvic region remains limited, primarily due to the difficulty in assembling a large multicenter cohort [13], [10]. Most of today’s DL-based synthesis methods are in 2D [5], requiring fewer patients in the training cohort than 3D learning. Additionally, 3D DL methods are less used due to their high computational cost [6].

DL methods can be categorized into two main groups: supervised and unsupervised. Supervised machine learning relies on labeled input and output training data, whereas unsupervised learning operates on unlabeled or raw data. Supervised generation requires very accurate non-rigid registration of the training cohort since MRI and CT cannot be simultaneously acquired to maintain consistent anatomical alignment [14]. This source of uncertainty underscores the need for unsupervised learning, which leverages ground truth data to extract valuable information and patterns, providing a promising solution to address this issue [15], [16]. An additional challenge lies in the significant complexity involved in creating a paired dataset, including the quantity of data, the precise delineation within both modalities, and achieving accurate registration [16]. To overcome the drawbacks of supervised methods, several unsupervised approaches have been proposed [5], [6], [17]. Indeed, unsupervised approaches do not require registered data. However, these unsupervised DL architectures encounter challenges related to non-convergence and instability attributed to the lack of an explicit loss function [18]. Despite the instability inherent in the absence of ground truth, this study proposes integrating an enhanced perceptual loss to improve convergence and facilitate the generation of more intricate images. In the context of sCT generation, the expression “Perceptual loss” (PL) (content term + style term) [19] refers to the use of the content term (a multi-scale similarity measure), which incorporates non-linear features [20]. In this study, the term “‘style” is adopted to impose constraints on the generator’s output, ensuring it produces CT-like styles.

The aim of this study was to develop and evaluate the performance of a 3D unsupervised conditional Generative Adversarial Networks (cGAN), in a multicenter context, compared to a supervised approach, for the purpose of generating prostate MR-to-CT images for RT dose calculation. A new perceptual loss was formulated using the ConvNext-tiny network [21], referred to as the Content and Style Representation for Enhanced Perceptual synthesis (CREPs) loss [19].

## 2 Material and methods

### 2.1 Image data

In this study, 99 patients treated for prostate cancer from three care centers (C1, C2, and C3) underwent one CT and MRI scan each (diagnostic MRI for C1 and C2, and MRI-Linac for C3) in the RT treatment position. C1 comprised 39 patients, with CT scans acquired using a GE LightSpeedRT large-bore scanner or a Toshiba Aquilion, and T2-weighted MR images acquired on a 3T Siemens Skyra MRI scanner [22]. C2 comprised 30 patients, with CT scans acquired on a Philips BigBore and T2-weighted MRIs on a 1.5T Siemens Skyra MRI scanner. Finally, C3 comprised 30 patients, with CT scans acquired on a GE LightSpeedRT16 and TrueFISP MRIs acquired on a 0.35T MRIdian (ViewRay) MRI-Linac. For all images, the target volume (prostate) and organs at risk (OARs, including bones (on the whole images), bladder, and rectum) were manually delineated by an expert. Ethics approval was granted by all hospitals, all patients gave written informed consent.

### 2.2 Experimental design

#### 2.2.1 Workflow of the study

**Fig. 1:**
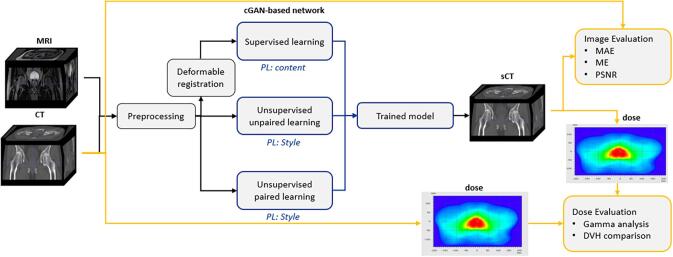
Workflow of the study. The workflow of synthetic computed tomography (sCT) generation is as follows: first, images were preprocessed; then, MRIs and CTs are non-rigidly registered for the supervised study. Afterwards, datasets are used to train three cGAN-based networks: a supervised one, an unsupervised unpaired one, and an unsupervised paired one. These networks use different computations of the perceptual loss (PL). Finally, sCTs are evaluated using Mean Absolute Error (MAE), Mean Error (ME), and Peak Signal-to-Noise Ratio (PSNR), and dose evaluation with gamma analysis and Dose Volume Histogram (DVH) comparison is performed.

The Fig. 1 presents the workflow of the study.

### 2.3 Image preprocessing

Several preprocessing steps were performed to standardize the database. First, the voxel intensities were clipped within the HU range of [−1000;3000] for CT scans, while values outside the range of [0;500] were excluded for MRIs. Following this, additional preprocessing steps were applied to correct the non-uniformity of the MRI images: (1) N4 bias field correction [23]; (2) histogram matching; and (3) filtering by gradient anisotropic diffusion [24]. Then, images (MRIs and CTs) were cropped at 12 cm below and above the barycenter of the prostate.

Deformable image registration was employed to align MRI and CT images [25]. Subsequently, a new cropping was performed to keep 8 cm above and under the prostate barycenter to obtain a common FOV for the entire image database. Finally, B-spline resampling was employed to apply dimensions of 256×256×128 for all images. Finally, CT/MRI pairs were divided into cohorts composed of 70 % of the data for the training, 10 % for the validation and 20 % for the test.

#### 2.3.1 Deep learning architecture

The DL architecture used in this study was a 3D cGAN built upon the Pix2Pix network backbone [26]. The generator ($G_{A}$), consisting of a 6-block ResNet, was used to convert MRIs into sCTs. The discriminator ($D_{A}$) was a 70×70×70 PatchGAN network, and the discriminator loss was the Binary Cross Entropy for all cases.

#### 2.3.2 CREPs loss

The Perceptual loss, using a pre-trained VGG model [19], is based on the concept of achieving independence between the style and content of an image. Our approach used this concept to synthesize a CT image from MRI content while preserving the CT’s style attributes. This loss function quantifies both the style and content by projecting the input image into a feature space tailored to capture either texture information or shape information, representing anatomy.

**Fig. 2:**
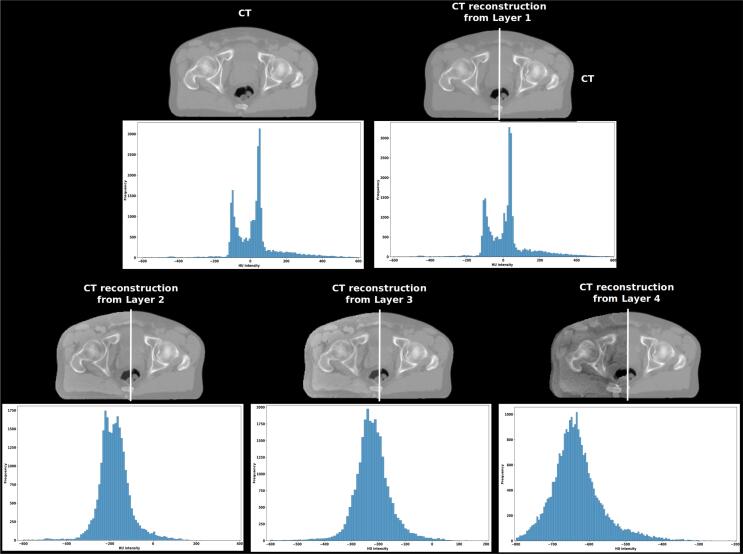
Representation of pelvis CT content extracted by inverse optimization. Similar to Mahendran et al. [27], an optimization to find an image y^ that minimizes the content reconstruction loss lcontϕ,j(.y^,y) is used.

To obtain a representation of the content, a pre-trained network for object recognition is employed. Networks trained for object recognition exhibit high sensitivity to the content of the image while remaining invariant to its precise appearance. This property becomes apparent during gradient descent between a white noise image and a target image. Fig. 2 illustrates how our image is reconstructed to retain the content but not the values. When the reconstruction is performed using the upper layers, the overall spatial structure is preserved, but not the exact values. Further details regarding the definitions of loss can be found in Supplementary Material 1 (S1). To address the computational limitations of the VGG-based PL in 3D, a novel Perceptual loss was formulated using the ConvNext-tiny [21] network referred to as the CREPs loss.

The ConvNext network is mainly based on the use of the ConvNext module of the same name. These modules were inspired by convolutional neural networks and Vision Transformer (ViT)[28] networks, which have outperformed other methods for image recognition tasks [21].

The ConvNext-Tiny network [21], which has fewer parameters, was used to construct a new Perceptual loss with a lower computational cost. The layers for the style and content loss functions were determined empirically.

#### 2.3.3 Supervised MR-to-CT method

In the supervised study, paired non-rigidly registered CT and MRI were used for the learning cohort. Paired data refers to cases where the CT and MRI images belong to the same patient. In this study, only the content term of the VGG-Perceptual loss [19] was used to compare the CT and sCT. In the medical synthetic image generation literature, the incorporation of style loss in the VGG-based perceptual loss definition has not been used [20].

#### 2.3.4 Unsupervised MR-to-CT method

Despite the discriminator’s assistance in adjusting the generation, it is observed that the learning process often suffers from non-convergence, especially in unsupervised training [29]. To ensure constrained output, the ConvNext-based CREPs loss [30] between sCT and CT was utilized to guide the unsupervised MR-to-CT synthesis. In this approach, only the style term of the perceptual loss was employed for unsupervised training. This decision stemmed from noticeable distinctions in content between MRI and CT modalities, making it difficult to achieve desired output by minimizing content loss from MRI. Incorporating the style term aims to enhance the stability of the learning process by constraining the solution space of the output. This constraint compels the network to extract common stylistic information from the CT, facilitating alignment between the output and the CT in the stylistic domain.

The unsupervised training approach for sCT generation has been explored through both unpaired (using a CT from one patient and the MRI from another patient) and paired training methodologies. In the unpaired scenario, the network was trained on CT and MRI data from two distinct patients, with patient selection randomized at each iteration within the training dataset.

### 2.4 Network implementation

The cGAN was implemented in Python 3.8 using Pytorch 1.12 with CUDA 11.7. The model was trained and tested on an NVIDIA RTX A6000 with 48 GB of VRAM. Trainings were performed on each center separately and on a multicenter cohort composed of a combination of the three centers. The tests were performed according to three scenarios: 1) a model trained on one center and tested on data from the same center (monocenter case), 2) a model trained on one center and tested on data from another center (unseen center case), and finally, 3) the multicenter model tested with data from the three centers (multicenter case). Supervised and unsupervised models were trained for 200 epochs, with a learning rate value 0.0001 and using Adam optimiser. The selection of the final model was based on the lowest MAE between the CT and sCT on the validation dataset during the training. To assess the robustness of these models, a 3-fold cross validation was performed for all the trainings.

### 2.5 Dose calculation

A treatment of 60 Gy (in 20 fractions) was planned for the PTV (prostate with an isotropic margin of 5 mm) using 6 MV photon beams VMAT on the reference CT with RayStation v.12A TPS (RaySearch). Subsequently, the beam parameters were applied to the corresponding patient’s sCTs to recalculate the dose.

### 2.6 Evaluation of sCT

#### 2.6.1 Image evaluation

To evaluate the accuracy of each sCT compared to ground truth CT, voxel-wise metrics [5] were used: the mean absolute error (MAE) in Hounsfield units (HU), the mean error (ME) in HU and the peak-signal-to-noise ratio (PSNR) in dB, as decribed as follows: the mean absolute error (MAE) in HU

(1) $$\mathrm{MAE}=\frac{1}{n}\overset{n}{\underset{i=1}{\sum }}|{\mathrm{sCT}}_{i}-{\mathrm{CT}}_{i}|$$

the mean error (ME) in HU,

(2) $$\mathrm{ME}=\frac{1}{n}\overset{n}{\underset{i=1}{\sum }}{\mathrm{sCT}}_{i}-{\mathrm{CT}}_{i}$$

and the peak signal to noise ratio (PSNR) in dB.

(3) $$\mathrm{PSNR}=10\log_{10}(\frac{Q^{2}}{\mathrm{MSE}})$$

with MSE as the mean square error (L2 norm between sCT and CT), and Q the amplitude.

These metrics were computed on five different volumes: the body contour (whole pelvis), the prostate, the bladder, the bones and the rectum. Wilcoxon tests were performed to determine whether the results were significantly different or not.

#### 2.6.2 Dose evaluation

Dose endpoints were absolute mean dose differences and the D95 % difference for the prostate volume. 3D gamma analysis (criteria: local analysis, 1 %/1 mm, with dose threshold = 10 %) between dose distributions on CTs and sCTs was performed using Verisoft (PTW). In terms of dose evaluation, only sCTs generated through multicenter training were assessed.

## 3 Results

### 3.1 Qualitative results

**Fig. 3:**
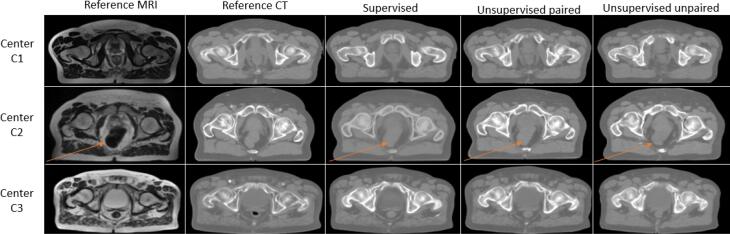
Image results with 3D cGAN according to the chosen learning process: supervised, unsupervised with paired data and unsupervised with unpaired data. The orange arrow shows the presence of gas in the rectum for MRI and the abscence of gas in the sCTs.

Fig. 3 shows the results of the cGAN-based MR-to-CT synthesis according to the context: supervised, unsupervised paired and unsupervised unpaired, using a multicenter training (from 3 different centers). The three methods provided visually realistic sCTs. It can be underlined that this algorithm do not reconstruct gas pockets.

### 3.2 Quantitative results

Table 1 summarizes the results of the quantitative analysis for sCT generation: MAE, ME, and PSNR values for the different multicenter learning methods (supervised generation, unsupervised unpaired, and unsupervised paired). On average, the MAE values for the body ranged from 33.6 HU to 45.5 HU across all test patients from the three centers. The supervised and unsupervised paired models showed similar performance, except for the bones. Additionally, soft tissues such as the prostate, bladder, and rectum exhibited lower MAE compared to bone tissue.

**Table 1:** Voxel-wise evaluation of multicenter sCT generation with the different learnings (supervised, unsupervised paired and unsupervised unpaired) on the different structures (External body, Bones, Prostate, Bladder and Rectum). Results in bold are the best ones according to the metrics. MAE results with a * represents the non significantly different (p value ≺ 0.0001) from the best result obtained on the organ.

$\frac{\mathrm{Metric}}{\mathrm{Architecture}}$	MAE (HU)	ME (HU)	PSNR (dB)	
	External body			
Supervised	37.4±10.3 *	7.5±17.7	25.8±2.2	
Unsupervised unpaired	45.5±12.4	-4.3±23.9	25.0±1.9	
**Unsupervised paired**	**33.6**±5.9 *	−**0.5**±**11.3**	**26.5**±**1.4**	
				
	Bones			
Supervised	159.4±60.1	89.7±109.1	15.9±2.6	
Unsupervised unpaired	114.6±27.3 *	9.1±49.4	18.1±2.2	
**Unsupervised paired**	**100.6**±27.2 *	**0.9**±**39.7**	**19.1**±**2.6**	
				
	Prostate			
Supervised	**27.5**±**31.5** *	8.5±37.6	20.7±4.2	
Unsupervised unpaired	32.4±25.2	**3.3**±**34.5**	21.5±4.1	
**Unsupervised paired**	**27.5**±23.1 *	5.6±28.7	**22.4**±**3.9**	
				
	Bladder			
**Supervised**	**26.6**±21.2 *	**1.2**±**30.2**	23.7±4.8	
Unsupervised unpaired	42.8±22.3	8.2±33.9	22.9±4.2	
Unsupervised paired	32.0±24.5 *	2.7±26.4	**25.0**±**5.0**	
				
	Rectum			
Supervised	39.7±32.3 *	-15.8±36.3	18.7±4.2	
Unsupervised unpaired	43.9±23.3 *	−**5.3**±3**4.5**	18.6±3.6	
**Unsupervised paired**	**34.3**±25.0 *	-9.9±25.2	**20.7**±**4.1**	

**Fig. 4:**
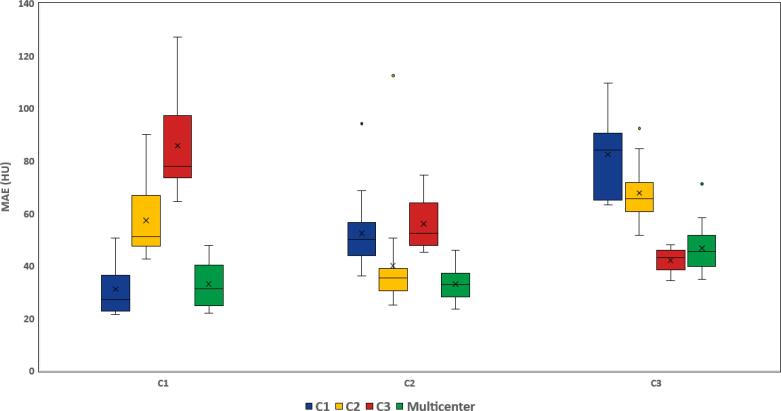
MAE results on external body: monocentric and multicentric supervised training comparison across centers. The boxplots illustrate the mean absolute error (MAE) between sCT and CT results for when the learning was performed on a cohort: C1 (blue), C2 (yellow), and C3 (red) and the three centers (green) and the test was performed on C1, C2 or C3.

Fig. 4 presents the differences in terms of MAE between reference CT and sCT generated from either monocenter or multicenter training in the supervised study. The best results were obtained for monocenter studies with seen data (reference) and multicenter training using the three centers in the learning cohort. Conversely, worse results were obtained when monocenter training was performed on one center and the test on another center.

### 3.3 Dose evaluation results

**Fig. 5:**
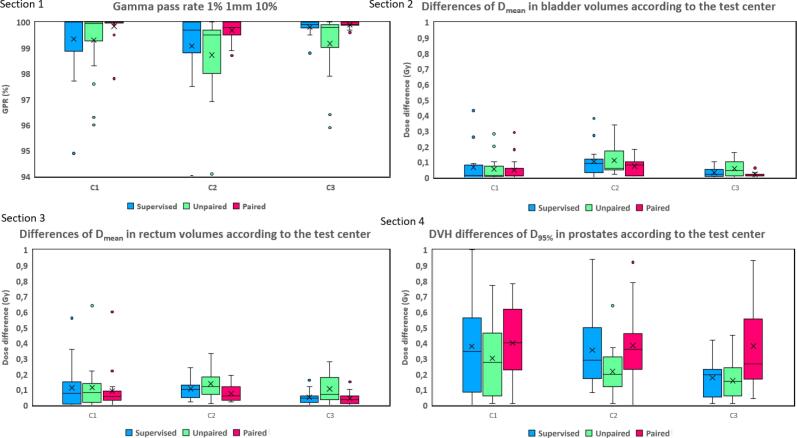
sCT dose evaluation results. Section 1 presents the results of the gamma pass rate (GPR) according to the method and the test center. Sections 2, 3 and 4 presents the DVH comparison (respectively absolute differences of mean dose in the bladder, absolute differences of mean dose in the rectum and absolute differences of D95 % in the prostate) results according to the methods (supervised in blue, unsupervised unpaired, in yellow, unsupervised paired, in pink) and the test center.

Fig. 5 presents dose evaluation results using multicenter trainings in this study. Section 1 displays gamma evaluation results, showing smaller mean gamma values for centers 2 and 3 under the unsupervised paired approach. However, overall mean gamma values consistently remained below the 0.75 threshold, with mean gamma pass rates over 94 % for all studies and over 98 % for the unsupervised paired method. Sections 2 to 4 detail DVH comparison evaluations. Absolute dose differences for OARs were under 0.7 Gy, with mean differences below 0.06 Gy for the bladder and 0.1 Gy for the rectum. The absolute difference of prostate D95 % between reference CT and sCT calculations was below 1 Gy, with an average absolute difference of 0.31 Gy.

## 4 Discussion

The results of this study highlight that accurate synthesis can be achieved in supervised contexts. However, unlike unsupervised approaches, the performance of this supervised MR-to-CT synthesis technique relies on registration accuracy. Table 1 highlights that bones and the rectum have the highest MAE. Gas pockets, which were not consistently reconstructed by deep learning algorithms [31], likely contribute to the variability in rectum MAE. Since modalities aren’t acquired simultaneously, gas might be present in one and not the other. Nevertheless, rectum dose assessment indicates an insignificant impact for VMAT treatments, with dose differences below 0.7 Gy in the entire rectum.

Moreover, it is underlined that regardless of the method (supervised, unsupervised paired, unsupervised unpaired), sCTs were accurate to allow dose calculation in clinics: the mean absolute dose difference were under 0.1 Gy for OARs and the gamma pass rates were above 94 % (with 1 %/1 mm criteria) for all patients. These results are comparable (even indirectly) to other literature results [5], [6], [32]. Contrary to MAE results, dose results were very close across the three methods. The Pearson correlation coefficient was computed between the mean gamma value and the MAE, revealing no linear correlation (≺ 0.3) between these two variables.

Furthermore, members of our team participated in the SynthRAD2023 Challenge [30] using a paired unsupervised cGAN approach. This challenge provides public multicenter datasets for the pelvis and brain regions, along with evaluation metrics for sCT generation algorithms. This facilitates a reliable comparison of performance among different generation methods. By employing the cGAN with CREPs loss, our results obtained in this challenge [30] for prostate sCT from MRI were a mean gamma pass rate of 98.5 % (criteria: local, 2 %/2 mm, threshold = 10 %) for photon irradiation. A significantly better performance in this study was observed when using paired data for both image and dose evaluations. This result was expected: the style term was closer in the case of paired data than in unpaired. Indeed, when the CT and MRI belonged to the same patient, the consistency was higher due to closer anatomical structures. This allowed the model to better capture the style information and generate more accurate sCT. However, results obtained with unpaired data were still satisfactory (gamma pass rate above 95 %), and this approach can still be useful when paired data with accurate deformable registration is not available. Furthermore, since the loss functions differed between the supervised and unsupervised studies, direct comparison of the results is not possible.

Multicenter training produces accurate results across multiple centers. Despite the diverse MRI systems ranging from 0.35T to 3T, comparable results were largely achieved due to preprocessing.

Since the MRI Linac was used for both planning and daily imaging, the same models can be employed. Therefore, the proposed multicenter sCT generation approach can also be extended to daily MRI for dose monitoring purposes. The concept behind this approach involves estimating the delivered dose by calculating the “dose of the day” on sCT and subsequently determining the cumulative dose, allowing for effective dose monitoring during the course of treatment. Our study has some limitations. It includes images from only 3 centers and more MRI scanners could be evaluated to broaden the models’ robustness. In addition there is no particular patient case which significantly differs from the training data (with a very high amount of adipose tissue, prosthesis, organ removal, etc.). In DL, the number and diversity of data are important in building robust methods.

In conclusion, this study presents an innovative unsupervised 3D cGAN leveraging a novel ConvNext-based Perceptual loss (CREPs loss) to generate sCT from MRI. The dosimetric results demonstrate that the unsupervised model performs comparably to supervised models, with no significant differences. The high-quality of the generated sCT images could eliminate actual CT scan simulation and CT/MRI registration from the standard RT workflow. This advance not only enhances patient care through workflow automation but also sets the stage for further clinical integration of MRI in RT.

## CRediT authorship contribution statement

**Blanche Texier:** Conceptualization, Methodology, Writing – original draft. **Cédric Hémon:** Conceptualization, Methodology, Writing – original draft. **Adélie Queffélec:** Methodology, Writing – review & editing. **Jason Dowling:** Writing – review & editing. **Igor Bessieres:** Data curation, Writing – review & editing. **Peter Greer:** Data curation, Writing – review & editing. **Oscar Acosta:** Resources, Writing – review & editing. **Adrien Boue-Rafle:** Data curation, Writing – review & editing. **Renaud de Crevoisier:** Resources, Writing – review & editing, Supervision, Funding acquisition. **Caroline Lafond:** Conceptualization, Writing – review & editing, Supervision, Project administration, Funding acquisition. **Joël Castelli:** Writing – review & editing, Supervision, Funding acquisition. **Ana**ï**s Barateau:** Conceptualization, Writing – review & editing, Supervision, Project administration, Funding acquisition. **Jean-Claude Nunes:** Conceptualization, Resources, Writing – review & editing, Supervision, Project administration, Funding acquisition.

## Declaration of competing interest

The authors declare the following financial interests/personal relationships which may be considered as potential competing interests: Cédric Hémon benefits from a PhD scholarship granted by Elekta AB.

## Supplementary data

upplementary data 1
