# Supplementary material for: 3D Unsupervised deep learning method for magnetic resonance imaging-to-computed tomography synthesis in prostate radiotherapy
Source: Phys Imaging Radiat Oncol. 2024 Jul 19;31:100612. doi: 10.1016/j.phro.2024.100612 (PMC11332181; doi:10.1016/j.phro.2024.100612)
Supplement: upplementary data 1 [file mmc1.pdf]

Supplementaty Material

6. CREPs loss details

Content loss function compared the feature maps of the two images extracted at different layers of the network. They are compared by the mean square error (Equation.4).

$$l^{\phi,j}_{cont}(\hat{I}_1, I_1) = ||\phi_j(\hat{I}_1) - \phi_j(I_1)||^2_2 \tag{4}$$

where $\phi_j(I_1)$ is the activation at the jth layer for the input I_1

To obtain a representation of the style of an input image, the feature space is constructed from the filter responses in any layer of the network. It consists of the correlations between the different filter responses taken over the spatial extent of the image. By including feature correlations from multiple layers, a stationary, multi-scale representation of the image is obtained, capturing texture information but not the overall arrangement. The style loss function does not directly compare the feature maps but rather the square Frobenius norm ($||...||^F_2$) between the Gram matrix Gram^{ϕ}_j of the output $\hat{I}_1$ (sCT) and the target I_2 (CT). The Gram matrix (Equation 5) captures information about characteristics that tend to activate together.

$$\text{Gram}^{\phi}_j(I_2) = \frac{\psi\psi^T}{C_j \times H_j \times W \times j} \tag{5}$$

$$\psi \text{ being the flattened matrix } \phi_j(I_2) \text{ of size: } C_j \times H_j \times W_j \tag{6}$$

$$l^{\phi,j}_{style}(\hat{I}_1, I_2) = ||\text{Gram}_j(\hat{I}_1) - \text{Gram}_j(I_2)||^F_2 \tag{7}$$
